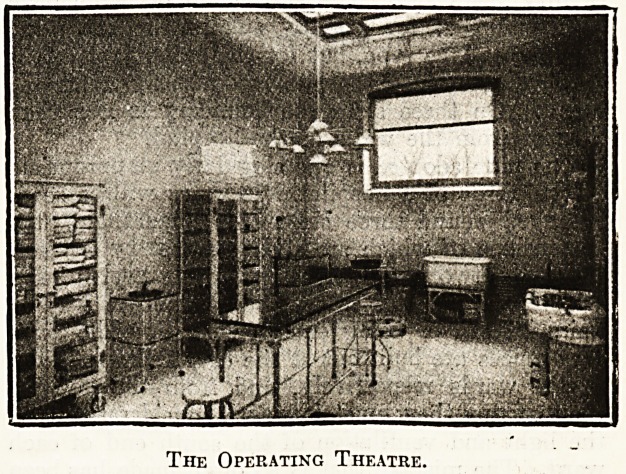# Some Interesting Institutions

**Published:** 1912-11-09

**Authors:** 


### SOME INTERESTING INSTITUTIONS.

(By Our Special Commissioner.)

### The Widener Cripple Institute, Philadelphia.

An easy tram-ride from the centre of the city of Philadelphia, along a fine wide avenue that leads to prettily wooded country on the outskirts of the city, brings the visitor to North Broad Street and Olney Avenue, the site of one of the most interesting orthopædic institutions in the world. Here, in grounds of thirty-two acres in extent, in an environment that is almost rural, stands the Widener Memorial Industrial Training School for Crippled Children, founded some five years ago by Mr. P. A. B. Widener, of Philadelphia, as a memorial to his wife and son, and erected at the cost of nearly £200,000, and endowed in perpetuity with a sum of £600,000. One of the most magnificent institutions of its kind in the world, the Widener School is a special hospital, an industrial colony, and a training institute at the same time, and a visit to its wards, cottages, and various special blocks will well repay the necessarily long time spent in inspecting the novel and interesting features of what may, in many respects, be taken as a model establishment.

The Institute from the Main Entrance.

### The Function of an Orthopædic Hospital.

Hospitals for cripples, orthopædic hospitals in the best sense of the term, are not mere establishments for the cure of certain specific conditions. It is in the vast majority of cases absurd to speak of a cure in the case of children afflicted with a disease which leaves permanent deformity behind it. The justification for the existence of special institutions to deal with such conditions lies in the fact that such children demand a very special, prolonged, and individual course of treatment—a course which cannot be given in a general hospital; which cannot, let it be added, be given in the majority of what are called orthopædic hospitals, or orthopædic departments of general hospitals, in this country, where no attempts are made to rival the completeness of the equipment and resources of foreign institutions. The valuable work which is carried on in these latter institutions must be seen to be thoroughly appreciated. In Russia, both at Moscow and St. Petersburg, the orthopædic hospitals have adjunct departments, which train and educate patients to become wholly self-supporting; a similar system has now been introduced in Italy (notably at the Instituto Rissoli and at Turin) and Germany, and in America it has long been considered as an essential, although in no institution has it been so thoroughly honoured as in the Widener Cripple School.

### Fallacious Comparisons.

Here the children are carefully taught how to utilise their often scanty resources of muscle and joint to enable them " to rise from dependency to independency, from helplessness to helpfulness." The usefulness of such social educative work can hardly be over-estimated, and anyone who wishes to see for himself the benefits of it should make a point of visiting the Treloar Home in this country and investigating the work done there. Necessarily the cost per patient rises considerably when this extra service is added to the routine methods of treatment which are already, in the case of orthopædic institutions, specially expensive, owing to the elaborate mechanical apparatus and the somewhat costly appliances required. It is therefore not fair to those institutions to compare them strictly with hospitals which do not supply social training. Nor is it just to compare them with sanatoriums where the patients do a certain amount of manual and other work: such work is often remunerative; it is not essentially, as is the case in these training hospitals, educative. When these few points are borne in mind it will be seen that there is justification for the high cost per patient which rules in these modern orthopædic clinics.

The Operating Theatre.

The routine at the Widener Institute is as follows: The institution is non-sectarian and is open to crippled children residing in the State of Pennsylvania or adjacent States. Candidates for admission must be between four and ten years old, this limit being absolutely necessary in order that early treatment and training may be followed with fair chance of success, and must be indentured, parents or guardians resigning all control over the child and binding it to the trustees until it reaches its majority. The child must be of good mental capacity, and must be a permanent cripple, though not necessarily a hopeless or absolutely helpless case. The candidate having been admitted, he or she is sent into the isolation building, an indispensable portion of the institution, which is simple and admirably adapted to its purpose. New arrivals are lodged here for two weeks before they are moved into the cottages or hospital building. If the child needs active hospital treatment, it is transferred to the ward, where it gets proper and prolonged orthopædic treatment.

### The Widener Treatment.

The treatment carried out is modern in every sense of the term, and amazingly thorough to those who are only accustomed to the slipshod methods still obtaining in our own so-called "orthopædic departments." When active treatment is no longer necessary, the child goes to a cottage and starts his education. He goes to an open-air school for two or more hours a day, getting a sound elementary education, with, later on, instruction in commercial subjects, such as typewriting, telegraphy, secretarial work, etc. From the earliest moment manual and industrial training is commenced. The following principles govern this training:—

The taste of a pupil for and his facility in any particular kind of work will largely influence the choice of trade or occupation.

Careful study of the physical ability and disability of each pupil is made, in order that there may be chosen for him a trade or occupation suited to his physical capacity for work. Other things being equal, the pupil is taught that trade that will develop and employ his greatest earning capacity. Trades paying higher wages are taught in preference to those paying lower wages or profits. Advanced pupils are paid for their labour, and are charged for board. An interest-bearing saving fund has been established, and the pupil is taught habits of careful accounting and of economy. As many of the graduate pupils as is practicable are employed in the institution as workmen in the buildings, on the grounds, and in the gardens; as maids and attendants in the hospital and cottages; as carpenters, as machinists and engineers, as instructors or undergraduates.

A dormitory has been erected for pupils who have completed their training, and who have secured employment in the city, but have no homes wherein to live. Selection is made from the following trades and occupations: Farming and gardening, floriculture, care of poultry and stock, dairying, carpenter work of all kinds, basket making, use of machines (brace making, engineering, ornamental iron work, etc.), leather work (making of shoes, covering of braces, etc., tailoring, dressmaking and millinery, printing and bookbinding, knitting and weaving, domestic arts (cooking, baking, housekeeping, etc.), engraving on silver or wood, upholstering.

Nothing can be more encouraging than to observe the way in which the children apply themselves to their work. The institute is a huge home: the best of relations exist between the resident staff and the children, and there is none of that "institutional shyness" which is so marked a characteristic in industrial school pupils. Our Commissioner was kindly shown over the buildings by an old house officer, and it was interesting to notice the demonstrative way in which this former superintendent was received by the children, the older ones of whom remembered him personally, while to the newcomers he was a hero and friend whose reputation had not suffered through more intimate acquaintance. The system of co-education has so far succeeded admirably. Discipline is easily maintained and the copartnership between boys and girls reminds one of the success that has been attained in the well-known mixed boarding-school in this country.

### The Buildings.

Designed by Mr. Horace Trumbauer, with the help of detailed suggestions by the staff orthopædists, who carefully considered the plans of modern orthopædic institutes, the buildings are worthy of the work which is carried on in them and of the munificence of the founder. The following is the detailed description, for which we are indebted to the official report:—

A large central hospital building, flanked by four separate cottages, two on the north and two on the south, which are connected with the central portion by covered passageways with movable sides of glass to be opened during suitable weather.

The main hospital building (Colonial style—brick with cement dressings) contains in the basement a large kitchen, apartments for the maids, office and supply rooms for the housekeeper, and a number of storage rooms. The food is conveyed from the kitchen to the several dining-rooms by a system of dumb-waiters.

The first floor contains in its central part the offices, reception rooms, examining rooms, drug store, dentist's office, lavatories and supply closets. The north wing is entirely occupied by a large dining-room for the children. In the south wing is the gymnasium, which is fully equipped with apparatus and machines for the correction of the various deformities of the patients. (This is indeed one of the finest orthopædic gymnasiums we have ever seen.)

On the second floor, in the south wing, is the ward for girls, with a large solarium on the extreme south end accommodating thirty-four children. A similar ward and solarium for boys occupies the north wing. The central part of the second floor is occupied by two smaller wards for acute medical cases and for post-operative cases, together with a surgical dressing-room, a special room, lavatories, linen closets, and a pantry.

On the third floor is a suite of four rooms used for surgical operations. Two other rooms are completely equipped for photographic work. Another is furnished with all the instruments necessary in the examination and treatment of the eye, ear, nose, and throat. There is an *x*-ray department, with a 24-inch spark machine; a dressing-room with shower bath and lavatory for the surgeons; and finally, there are storage rooms and closets.

An elevator, large enough to accommodate beds and rolling chairs, runs from the basement to third floor. There is an elevator, also, in each cottage building for the use of those children that cannot walk up and down the stairs.

The cottages for the children, one for the boys and one for the girls, are situated to the north of the main building. On the first floor of each cottage there are well-furnished library, dining and play-rooms, with an office room for the house-mother. On the second floor is a dormitory, containing twelve beds for small children. In addition there are ten private rooms for older children, together with a room and a private bath for the house-mother.

The industrial building is situated to the south-west of the central building. It is designed to be occupied by the various departments of the industrial school, none of which have yet been organised. At present it contains the machine shop and forge room, where are made all the braces used by the patients. A skilful brace-maker resides in the school and gives all his time to this work. On the same floor is a well-equipped laboratory, where the resident physician may make any of the ordinary tests and reactions used in medical practice.

The second floor contains the school rooms, one for the kindergarten and two for the school proper. The former is furnished with the various apparatus employed in kindergartens. In the latter there are seats and desks that may be adjusted to suit the individual deformity of each child.

In the basement and sub-basement of this building is the power plant. Here are generated the heat, the electric light, and the electro-motive power for the entire institution. There is also an ice-manufacturing plant.

### Some Structural Details.

The corridors and hallways are heated by steam radiators; the wards and rooms by air drawn fresh from without and conducted over heated steam pipes in the basement. The ventilation and heating of the institute are almost perfect: at the time of our visit no closeness or mustiness was to be detected anywhere, and the passages were entirely free from the school smell which is so familiar to those who have worked in rooms where children congregate.

The administration block, to the south-east of the central building, contains the offices and quarters of the residential staff. A matron, resident medical officer, and several specially-trained nurses reside here. The isolation building has a north and south wing, which can be maintained separately without intercommunication, and lies to the west quite apart from the main group. At the time of our visit there were sixty-four children in residence. Our thanks are due to the officers of the institution for their courtesy, and especially to Dr. Merrill, of Philadelphia, for his personal guidance over the institute.

## Figures and Tables

**Figure f1:**
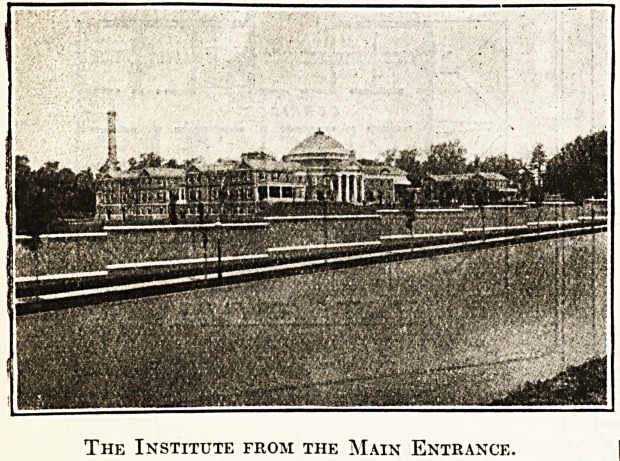


**Figure f2:**